# Chiropractic care for children: too much, too little or not enough?

**DOI:** 10.1186/1746-1340-18-17

**Published:** 2010-06-02

**Authors:** Simon D French, Bruce F Walker, Stephen M Perle

**Affiliations:** 1 Primary Care Research Unit, The University of Melbourne, Australia; 2 Australasian Cochrane Centre, School of Public Health and Preventive Medicine, Monash University, Australia; 3 School of Chiropractic and Sports Science, Faculty of Health Sciences, Murdoch University, Australia; 4 College of Chiropractic, University of Bridgeport, USA

## Abstract

This editorial provides an overview of this Thematic Series of the journal titled Chiropractic Care for Children. In commissioning this series of articles we aimed to bring the busy clinician up to date with the current best evidence in key aspects of evaluation and management of chiropractic care for children. Individual articles address a chiropractic approach to the management of children, chiropractic care of musculoskeletal conditions in children and adolescents, chiropractic care of non-musculoskeletal conditions in children and adolescents, chiropractic care for attention-deficit/hyperactivity disorder and possible adverse effects from chiropractic management of children. The final article by Charlotte Leboeuf-Yde and Lise Hestbæk is an overview of the current state of the evidence and future research opportunities for chiropractic care for children. We conclude this editorial discussing the strengths and weaknesses of contemporary research relevant to chiropractic care of children and the implications for chiropractic practice.

## Background

Many chiropractors provide care to children and chiropractors treat a wide variety of paediatric health conditions [1]. This is considered a controversial area of chiropractic management, both within [2,3] and outside of the profession [4,5]. Within the profession, there has been a recent call for chiropractors to assume the responsibility of spinal and musculoskeletal health in children [6]. Evidence is mounting that childhood health and lifestyle may have an impact on health and quality of life in later years, that chiropractors provide care to children and cannot be ignored [6]. The evidence-base for chiropractic care for children is scarce, however some evidence is available to inform practice. In commissioning this thematic series for *Chiropractic & Osteopathy*, we have brought together key people in the field of chiropractic care for children to provide an up-to-date overview for clinicians and researchers interested in the role of chiropractic care for children.

## Discussion

The management techniques that chiropractors employ for children vary across the profession [1], but typically they are techniques modified from those used for adult patients. Although spinal manipulative therapy in its many forms is a core part of a chiropractor's treatment approach, the term "chiropractic care" in relation to this thematic series refers to the entire chiropractic clinical encounter which may also include other treatments such as dietary advice, nutritional or herbal supplements, posture correction, exercise prescription, physiotherapeutic modalities and behavioural counselling [2]. The series of articles we have commissioned for this topic have focussed on the manual therapies that chiropractors deliver.

### The chiropractic approach to the management of children

The first article in this thematic series presents a chiropractic approach to the management of the paediatric patient and makes recommendations as to how the chiropractic profession can safely and effectively manage the paediatric patient [7]. It also provides an overview of current chiropractic education in paediatric management. The authors conclude that there is little research on which to base current practice, and that the chiropractic profession needs to improve this evidence base in the interest of what is best for the paediatric population who present to chiropractors in practice.

### Chiropractic management of musculoskeletal conditions in children and adolescents

The interventions chiropractors use are supported in part by the evidence-base for manual therapies for some musculoskeletal conditions, particularly low-back pain [8-12]. However, this evidence base is solely in the setting of musculoskeletal conditions in adults. The second article in this series is a systematic review of the evidence for chiropractic care of musculoskeletal conditions in children and adolescents [13]. Low back pain is common in children and adolescents [14], but high quality evidence for chiropractic management, and even more broadly for manual therapies, of musculoskeletal conditions in children is simply non-existent. If the chiropractic profession is to assume some sort of authority for the care of children's musculoskeletal health, appropriate and high quality research must be urgently undertaken to determine what type of chiropractic care is appropriate.

### Chiropractic diagnosis and management of non-musculoskeletal conditions in children and adolescents

This overview discusses and summarises the literature about diagnosis and management of non-musculoskeletal conditions in children and adolescents [15]. The authors conclude that the more scientifically rigorous studies show conflicting results for chiropractic care for colic and the crying infant, and that there is little data to support or refute the effectiveness of chiropractic care for otitis media, asthma, nocturnal enuresis or attention deficit hyperactivity disorder. The authors do recommend that a chiropractor may play a role in the paediatric healthcare team. They suggest that, despite the conflicting evidence, a trial of four to six chiropractic visits are reasonable for a colicky infant where all other serious diagnoses have been excluded. For enuresis and asthma the authors suggest that the chiropractor may have a role in a multidisciplinary approach addressing part of the clinical picture. Repeating a common theme through this series of articles, these authors call for more research to be conducted relevant for the chiropractic management of non-musculoskeletal conditions.

Their recommendations are somewhat controversial as they advocate a role for chiropractic where the evidence is less than satisfactory. We believe that caution needs to exercised where evidence exists against a modality. It does not serve patients, or the chiropractic profession, well to provide treatment that has been shown to be ineffective or where there is insufficient evidence to reach a conclusion when there are other options available that have demonstrated benefits [16].

### Chiropractic care for paediatric and adolescent attention-deficit/hyperactivity disorder

A more focussed systematic review examines the evidence-base for chiropractic care for attention-deficit/hyperactivity disorder (AD/HD) in children. The evidence comes up short with no identified studies meeting the authors' inclusion criteria. The authors conclude that the claim that chiropractic care improves paediatric and adolescent AD/HD is only supported by low levels of evidence. They then go on to discuss specific research that can be undertaken to address this lack of evidence.

### Possible adverse effects of chiropractic management of children

This article provides a review of possible adverse events in children treated by manual therapy [17]. The author concludes that there is currently insufficient research evidence related to adverse events and manual therapy, but that this therapy appears only to cause mild to moderate adverse events which are common and self limiting. Serious adverse events in children undergoing manual therapy are rare. This author also calls for more high quality research in this area, specifically addressing adverse events and paediatric manual therapy.

### Future research opportunities for chiropractic care for children

Our final article in this thematic series addresses the question "Is more research enough?" [18]. The authors tackle this question by proposing that more research in this area is not enough, in that research needs to be appropriate and of high quality. They discuss both the lack of evidence in general in the area of chiropractic care for children, and also the lack of research using appropriate study designs. In particular, they suggest that low levels of evidence, for example case reports purportedly demonstrating therapeutic benefit, should not be conducted because they have no value in judging the effects of therapies.

### What sort of further research is needed in this area?

As consistently demonstrated in the review articles in this Thematic Series, effective chiropractic management of children is not supported by strong evidence, but chiropractic care for children seems to carry a very low risk of adverse events. More appropriate and high quality research is needed to examine chiropractors' role in the management of children and their health conditions. The "more research is needed" statement is seen in so many reviews across many healthcare fields and is not unique to chiropractic care. However, the responsibility lies with the profession who claims to offer effective treatment, and in the case of chiropractic care of childhood conditions, the evidence is consistently lacking.

The type of research that is needed is briefly covered in the commentary by Leboeuf-Yde and Hestbaek in this series [18], but we would add to this. High quality research does not come cheaply and funds must be spent wisely. The chiropractic profession needs a concerted effort to determine what the current research priorities are for the profession and actively engage the research community to carry out this research. In the first instance, high quality observational research is needed to determine what type of paediatric patients are presenting to chiropractors and what type of care is being offered. We have very little information about who seeks chiropractic care, why these people seek care and what type of care is provided. There is also currently no data on what percentage of children who have problems seek chiropractic care, and for which conditions this care is sought. In addition, more high quality effectiveness and safety research studies to determine the benefit and potential harms of chiropractic care for children is required. Finally, the management of childhood illnesses requires considerable skills in diagnosis as well as therapy. It is not clear whether all, or any, chiropractic curricula currently include sufficient training in paediatrics that would provide chiropractors with the depth and breadth of training required to make a diagnosis and carry out uniformly accepted therapy. This area is fertile for educational research.

### What is a clinician to do when no evidence exists?

Should chiropractors be accepting and treating children considering the scarce evidence available? Are chiropractors qualified to diagnose and treat children who present for their care? Should chiropractors charge money for treatment that does not have evidence to support that it is effective?

Considering the evidence presented in this thematic series, and other evidence, some key issues need to be addressed for members of the chiropractic profession in relation to the care of children. We believe there are a number of issues chiropractors should consider before they provide care to a child or infant who presents to them.

Given the current poor state of the evidence presented here in the articles in this thematic series, and in other related articles [2,19], should chiropractors be treating children at all? Evidence-based practice provides guidance for clinicians to make clinical decisions with individual patients when strong evidence is not available. Guided by clinical experience and patient preferences, the chiropractor and their patient (and parent) can make an informed choice about the use of chiropractic care for a child patient.

For some childhood conditions discussed in this thematic series, for example excessive crying and infant colic [15], there is currently no other effective treatment available. Some people suggest, including the authors of the paper in this thematic series addressing non-musculoskeletal conditions [15], that it is reasonable that a short trial of chiropractic care is considered. As researchers, we caution against clinicians accepting this suggestion without question. There is no evidence that chiropractic care for infant colic is more effective than sham therapy [19]. Thus it may also be reasonable to suggest that a short trial of "placebo treatment" is warranted! With the current state of the evidence, it is difficult to recommend a trial of chiropractic care, as opposed to other treatments with no proven effect.

The chiropractor should reflect on their training, both undergraduate and postgraduate, and decide whether they are qualified to make a diagnosis for a child's condition, and subsequently whether they have the skill to provide appropriate chiropractic care for children. The management of childhood illnesses requires considerable skills in diagnosis and therapy. If the chiropractor has any doubt about their clinical capability after considering this issue, a close relationship with another healthcare professional who has more appropriate qualifications and skill may be a useful model to provide shared care of the a child. We would suggest that given the population we are speaking of, children, chiropractors should be very self-critical of their clinical capabilities. Of course, if the chiropractor considers that the presenting condition is outside the scope of their practice, they should refer the patient to the appropriate healthcare professional for care.

An open dialogue with patients and their parents is essential. When a chiropractor considers that a trial of treatment is warranted but no evidence exists for a given treatment, or there is evidence that the treatment is no more effective than placebo as for infant colic [19], patients and their parents should be informed of this. This ensures that the "patient preferences" arm of the evidence-based triangle is addressed [20]. A joint decision-making process between chiropractor, the patient and the parent/s can only lead to better outcomes for all involved.

Finally, all chiropractors who treat children should be adopting current best practice as proposed by the chiropractic profession itself. Chiropractic care for children was the subject of a recent consensus process, and chiropractors should be aware of this document and the recommendations contained within it [2]. This document provides a general framework for what constitutes an evidence-based and reasonable approach to the chiropractic management of infants, children, and adolescents. It addressed issues such as informed consent, sole and co-management, how to conduct a clinical history, red flags in a paediatric patient, diagnostic imaging and manual treatment.

## Conclusions

Lamenting the lack of an adequate base of good quality research is nothing new in chiropractic. In the 1930s and 1940s, C.O. Watkins asked the profession to step up and produce research [21]. In 1975 a landmark workshop on the research status of spinal manipulation was conducted by the United States National Institutes of Health's National Institute of Neurological Disorders and Stroke [22]. This workshop might be seen as the starting point for the science of spinal manipulation. The workshop produced a call for more research. Since then further calls for more work have come from various quarters. We shall do the same.

As these series of articles suggest, there is currently little evidence to inform chiropractic care of children. The chiropractic profession needs to be responsible for moving forward the evidence-base from which to inform chiropractic clinical practice for children. We suspect that no other profession will do this for us!

## Competing interests

The three authors are part of the Editorial Team for Chiropractic & Osteopathy. Otherwise, the authors declare that they have no competing interests.

## Authors' contributions

SDF wrote the first draft of the manuscript and BFW and SMP contributed significant editorial input. All authors have read and approved the manuscript.
